# Increased frequency of IgD-CD27^hi^CD38^hi^ B cells and its association with the renal involvement in ANCA-associated vasculitis

**DOI:** 10.1186/s13075-022-02796-9

**Published:** 2022-05-14

**Authors:** Chen Wang, Zhi-Ying Li, Yan Gong, Ran You, Ming-Hui, Zhao, Min Chen

**Affiliations:** 1 Renal Division, Department of Medicine, Peking University First Hospital; Peking University Institute of Nephrology, NO.8 Xishiku Street, Xicheng District, Beijing, 100034 China; 2 Key Laboratory of Renal Disease, Ministry of Health of China, Beijing, China; 3 Key Laboratory of Chronic Kidney Disease Prevention and Treatment (Peking University), Ministry of Education, Beijing, China; 4 Department of Clinical Laboratory, Peking University First Hospital, Beijing, China; 5 Peking-Tsinghua Center for Life Sciences, Beijing, China

**Keywords:** Antineutrophil cytoplasmic antibody, Vasculitis, Renal involvement, B cells

## Abstract

**Background:**

B cells have been highlighted in the pathogenesis of antineutrophil cytoplasmic antibody (ANCA)-associated vasculitis (AAV) by the identification of activated B cells in granulomatous lesions and the efficacy of B cell depletion in treatment of AAV patients in the current study; we aimed to investigate the frequency of a specific B cell subset, IgD-CD27^hi^CD38^hi^ B cells in AAV patients, and its association with the disease severity of AAV.

**Methods:**

Blood samples of patients with AAV in active stage and in remission were collected. The frequency of IgD-CD27^hi^CD38^hi^ B cells was detected by flow cytometry, and its correlation with clinicopathological parameters was analyzed.

**Results:**

Our results showed a significant increase of circulating IgD-CD27^hi^CD38^hi^ B cells in AAV patients in active stage compared with patients in remission and healthy donors, and the frequency of IgD-CD27^hi^CD38^hi^ B cells correlated with the severity of renal involvement, including serum creatinine, estimated glomerular filtration rate, and percentages of total crescents in renal biopsies.

**Conclusions:**

The results indicated that IgD-CD27^hi^CD38^hi^ B cells could reflect disease severity of renal involvement in AAV.

**Supplementary Information:**

The online version contains supplementary material available at 10.1186/s13075-022-02796-9.

## Introduction

Antineutrophil cytoplasmic antibody (ANCA)-associated vasculitis (AAV) is a group of severe autoimmune disorders that commonly involve the kidney. It consists of three distinct clinical entities, i.e., granulomatosis with polyangiitis (GPA, previously named Wegener’s granulomatosis), microscopic polyangiitis (MPA), and eosinophilic granulomatosis with polyangiitis (EGPA) [1]. The histopathological hallmark of ANCA-associated glomerulonephritis is pauci-immune necrotizing crescentic glomerulonephritis [2]. ANCA is the serological marker of AAV, predominantly immunoglobulin (Ig)G class autoantibody against primary granule constituents of neutrophils and lysosomes of monocytes, in particular, myeloperoxidase (MPO) and proteinase 3 (PR3) [3].

Despite the pathogenesis of AAV has not been fully elucidated, the pathogenic role of ANCA has been well demonstrated by clinical observations, animal studies, and in vitro studies [4–8]. Furthermore, as the precursors of antibody-bearing cells, B cells have been highlighted in the AAV pathogenesis by the identification of activated B cells in granulomatous lesions and the efficacy of B cell depletion in treatment of AAV patients [9, 10].

B cells can be subdivided into naïve cell (CD27-IgD+), pre-switched memory cell (CD27+IgD+), conventional memory cell (CD27+IgD-), double-negative (DN) memory cell (CD27-IgD-), plasmablast (CD27+CD38^hi^IgM-), and plasma cell (PC, CD27+CD138+) subsets according to their surface markers [11, 12]. Altered B cell subset distribution was observed in several autoimmune diseases, including AAV [13–16]. In autoimmune diseases, among various B cells, autoreactive B cells represent the main effector B cells in pathogenic conditions of autoimmunity. However, the definition, pathophysiological feature and development process of autoreactive B cells remain unknown. Tipton et al reported that circulating IgD-CD27^hi^CD38^hi^ B cells in patients with systemic lupus erythematosus (SLE), which increased up to 40-fold compared with normal controls, contributing substantially to the serum autoantibody repertoire in SLE [17]. Pozdzik et al suggested that circulating CD20-IgD-CD27^hi^CD38^hi^ B cells was a new cellular biomarker of residual autoimmunity in anti-PLA2R1 related membranous nephropathy [18]. In AAV, von Borstel et al found an increased frequency of circulating CD27 + CD38^hi^ B cells during remission, which was associated with a higher relapse risk in GPA patients [19]. In their study, whether there was an association between the frequency of CD27 + CD38^hi^ B cells with disease activity of GPA did not been fully investigated.

Taken together, the above-mentioned studies indicated a promising role of such a specific B cell subset of switched memory B cells or plasmablasts, which might be closely related to the autoreactive B cells, in the pathogenesis of autoimmune diseases, including AAV. Therefore, it is of interest to further investigate the frequency of IgD-CD27^hi^CD38^hi^ B cells in patients with active AAV and its association with the severity of renal involvement and disease activity of AAV patients.

## Methods

### Patients and samples

Twenty-six patients with AAV in active stage, diagnosed at Peking University First Hospital from July 2014 to September 2015, were recruited in this study. Fourteen out of these 26 patients with active AAV received renal biopsy. Eighteen patients with AAV, who achieved complete remission after immunosuppressive therapy, were also recruited at their regular ambulatory visits. Treatment protocols were described previously [20]. In brief, patients received the induction therapy typically including corticosteroids in combination with cyclophosphamide (CTX). Patients with severe pulmonary hemorrhage or acute renal failure requiring dialysis at diagnosis received additional plasmapheresis. For maintenance therapy, started after induction therapy, daily oral azathioprine (AZA, 2 mg/kg/day) was given. “Remission” was defined as “absence of disease activity attributable to active disease qualified by the need for ongoing stable maintenance immunosuppressive therapy” (complete remission), or “50% reduction of disease activity score and absence of new manifestations” (partial remission), as described previously [21]. Blood samples from all these patients were collected. Among the AAV patients mentioned above, there were eight patients who had blood samples both in active stage and remission. All the patients met the Chapel Hill Consensus Conference (CHCC) definition of AAV [1]. Patients with secondary vasculitis, cancers, or with comorbid renal diseases, such as IgA nephropathy or lupus nephritis were excluded. Disease activity was assessed according to the Birmingham Vasculitis Activity Score (BVAS) [22]. Blood samples of five age- and gender-matched healthy blood donors were collected as the normal controls. We obtained written informed consent from each participant involved in our study. The research was in compliance with the Declaration of Helsinki and was approved by the clinical research ethics committee of the Peking University First Hospital.

### Detection of ANCA

ANCA test was performed by antigen specific enzyme-linked immunosorbent assay (ELISA). Standard ELISA assays for MPO-ANCA and PR3-ANCA were performed according to the manufacturer (EUROIMMUN, Lubeck, Germany).

### Renal histology

Renal histology was evaluated according to the previous standardized protocol [23–26]. The presence of glomerular lesions, including fibrinoid necrosis, crescents, and glomerulosclerosis, was calculated as the percentage of the total number of glomeruli in biopsy findings. Interstitial and tubular lesions were scored semiquantitatively on the basis of the percentage of the tubulointerstitial compartment that was affected as following: interstitial infiltrate (“−” for 0%, “+” for 0–20%, “++” for 20–50%, and “+++” for  > 50%), interstitial fibrosis (“−” for 0%, “+” for 0–50%, and “++” for > 50%), and tubular atrophy (“−” for 0%, “+” for 0–50%, and “++” for > 50%).

### Flow cytometric analysis

**Fig. 1:**
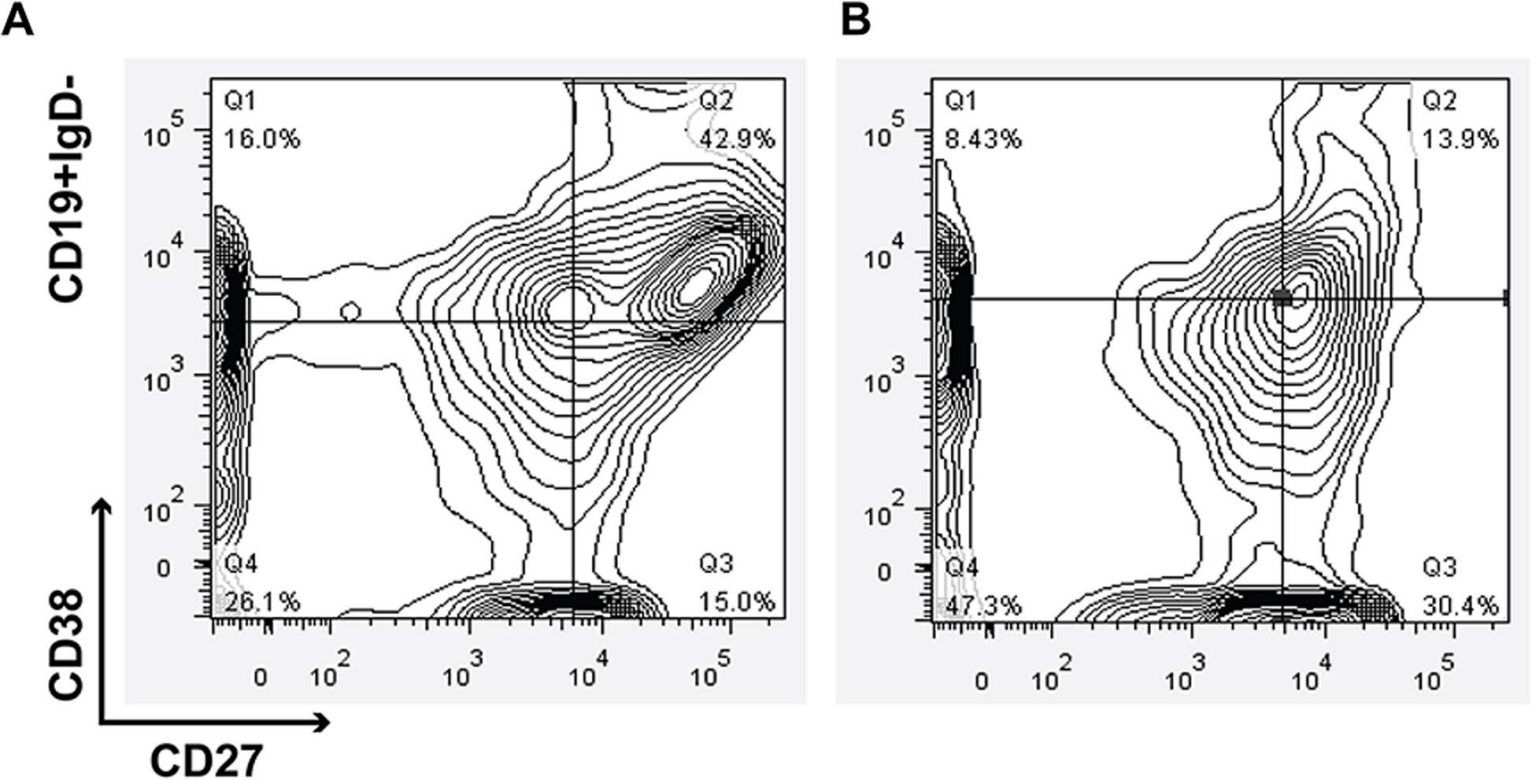
A representative gating example of sequential blood sample from an AAV patient. **A** The distribution of IgD-CD27^hi^CD38^hi^ B cells in the patient in active stage. **B** the distribution of IgD-CD27^hi^CD38^hi^ B cells in the patient in remission

Peripheral blood lymphocytes isolated from venous blood samples of AAV patients and healthy donors were analyzed by flow cytometry. A 100-μl sample of whole blood was washed and stained with APC-Cy7-CD45, PerCP-CY5.5-CD19, PE-CY7-IgD, APC-CD27, and BV421-CD38 (BD, San Diego, USA) at room temperature for 30 min. Isotype-matched antibodies were used as negative controls. Erythrocytes were lysed using a commercially available lysing solution (BD, San Diego, USA). The cells were washed twice and resuspended in 0.5 ml of phosphate-buffered saline (PBS). Labeled cells were acquired on a FACSCanto II flow cytometer and were analyzed with FACSDiva software (BD, San Diego, USA). Figure 1A, B shows a representative gating example of sequential blood sample from an AAV patient.

### Statistical analysis

Quantitative data were expressed as the means ± SD (for normally distributed data) or as the medians and ranges or quartiles (for non-normally distributed data) as appropriate. Correlation analyses were performed using Pearson’s test (for normally distributed data) or Spearman’s rank test (for non-normally distributed data). Differences of quantitative parameters between groups were assessed using *t-*test, one-way ANOVA (for normally distributed data), or the Mann-Whitney *U* test (for non-normally distributed data) as appropriate. Differences were considered significant when *p* < 0.05. The analysis was performed with the SPSS statistical software package (version 17.0; Chicago, IL).

## Results

### General data of the patients

Among the 26 AAV patients, 15 were male, and 11 were female, with an age of 61.1 ± 12.3 years at diagnosis. Twenty-three patients were MPO-ANCA positive, and 3 patients were PR3-ANCA positive. The median duration between symptomatic disease and diagnosis was 3 months (range 0.5-45). The level of BVAS was 17.4 ± 4.8. The level of initial serum creatinine was 481.8 ± 246.9 μmol/l. The general information for these patients is listed in Table 1.

**Table 1:** Clinical and histopathologic data of patients with active AAV

General clinical data	Number (%)
Number	26
Male/female	15/11
Age (years)	61.1 ± 12.3
MPO-ANCA/PR3-ANCA	23/3
The duration between symptomatic disease and diagnosis (months)	
Median	3.0
Range	0.5–45.0
Scr (μmol/L)	
Mean ± SD	481.8 ± 246.9
Range	106.7–955.0
eGFR (ml/min/1.73m^2^)	
Mean ± SD	14.6 ± 10.7
Range	4.4–47.6
Urinary protein (g/24 h)	
Median	0.71
Range	0.11–8.31
BVAS	17.4 ± 4.8
Renal pathological data	
Number	14
Normal glomeruli (%)	35.6 ± 20.9
Glomerular lesions (%)	
Total crescents	59.2 ± 23.6
Cellular crescents	52.9 ± 21.5
Fibrous crescents	6.3 ± 12.3
Fibrinoid necrosis	3.2 ± 3.1
Global sclerosis	2.0 ± 3.4
Tubulointerstitial lesions	
Interstitial infiltration (−/+/++/+++)	0/2/10/2
Interstitial fibrosis (−/+/++)	1/2/11
Tubular atrophy (−/+/++)	1/10/3

*Abbreviations*: *BVAS* Birmingham Vasculitis Activity Scores, *Scr* serum creatinine, *eGFR* estimated glomerular filtration rate, *SD* standard deviation

### Increased frequency of IgD-CD27^hi^CD38^hi^ B cells in patients with active AAV

**Fig. 2:**
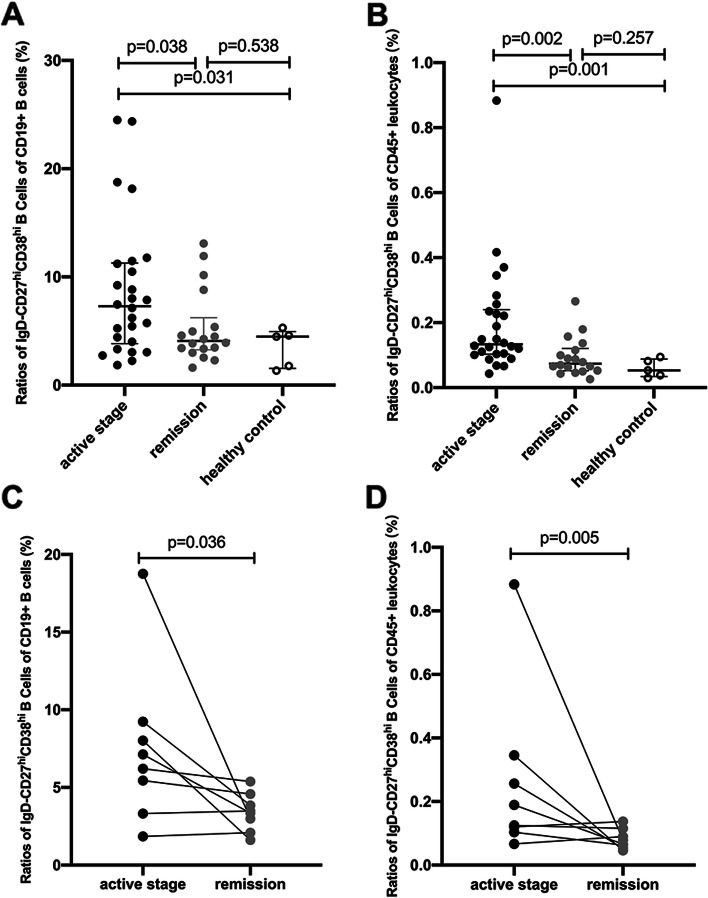
Increased frequency of IgD-CD27^hi^CD38^hi^ B cells in patients with active AAV. **A** The frequency of IgD-CD27^hi^CD38^hi^ B cells among CD19+ B cells was significantly increased in the AAV patients in active stage than those in both AAV patients in remission and healthy donors. **B** The frequency of IgD-CD27^hi^CD38^hi^ B cells among CD45+ leukocytes was significantly increased in the AAV patients in active stage than those in both AAV patients in remission and healthy donors. **C** The frequency of IgD-CD27^hi^CD38^hi^ B cells among CD19+ B cells was significantly increased in active stage than that in remission in 8 AAV patients with sequential blood samples of both active stage and remission. **D** The frequency of IgD-CD27^hi^CD38^hi^ B cells among CD45+ leukocytes was significantly increased in active stage than that in remission in 8 AAV patients with sequential blood samples of both active stage and remission

The frequency of IgD-CD27^hi^CD38^hi^ B cells was compared between the AAV patients in active stage and remission as well as control groups. The frequency of IgD-CD27^hi^CD38^hi^ B cells among CD19+ B cells was significantly higher in AAV patients in active stage than those in AAV patients in remission and healthy controls (median 7.29% [IQR 3.84–11.28%] vs. median 4.08% [IQR 3.26–6.23%], *p* = 0.038; median 7.29% [IQR 3.84–11.28%] vs. median 4.49% [IQR 1.55–4.95%], *p* = 0.031, respectively). In parallel, the frequency of IgD-CD27^hi^CD38^hi^ B cells among CD45+ leukocytes was significantly higher in AAV patients in active stage than those in AAV patients in remission and healthy controls (median 1.33‰ [IQR 1.03–2.40‰] vs. median 0.74‰ [IQR 0.52–1.21‰], *p* = 0.002; median 1.33‰ [IQR 1.03–2.40‰] vs. median 0.53‰ [IQR 0.34–0.88‰], *p* = 0.001, respectively). There was no significant difference in the frequency of IgD-CD27^hi^CD38^hi^ B cells between AAV patients in remission and in healthy controls (for IgD-CD27^hi^CD38^hi^ B cells among CD19+ B cells, median 4.08% [IQR 3.26–6.23%] vs. median 4.49% [IQR 1.55–4.95%], *p* = 0.538; for IgD-CD27^hi^CD38^hi^ B cells among CD45+ leukocytes, median 0.74‰ [IQR 0.52–1.21‰] vs. median 0.53‰ [IQR 0.34–0.88‰], *p* = 0.257, respectively, Fig. 2A, B).

Furthermore, we compared the frequency of IgD-CD27^hi^CD38^hi^ B cells in 8 AAV patients with sequential blood samples of both active stage and remission. Consistent with the abovementioned results, the frequency of IgD-CD27^hi^CD38^hi^ B cells was significantly higher in active stage than that in remission (for IgD-CD27^hi^CD38^hi^ B cells among CD19+ B cells, median 6.67% [IQR 3.85–8.93%] vs. median 3.42% [IQR 2.32–4.40%], *p* = 0.036; whereas, for IgD-CD27^hi^CD38^hi^ B cells among CD45+ leukocytes, median 1.57‰ [IQR 1.08–3.23‰] vs. median 0.74‰ [IQR 0.55–1.10‰], *p* = 0.05). Six out of these 8 patients had a decrease in frequency of IgD-CD27^hi^CD38^hi^ B cells in remission compared with that in active stage, whereas only 2 patients had a slight increase in frequency of IgD-CD27^hi^CD38^hi^ B cells in remission compared with that in active stage (Fig. 2C, D). Induction therapy of these 8 patients in active stage was listed as Supplementary Table.

### Association between the frequency of IgD-CD27^hi^CD38^hi^ B cells and severity of renal involvement in patients with active AAV

**Fig. 3:**
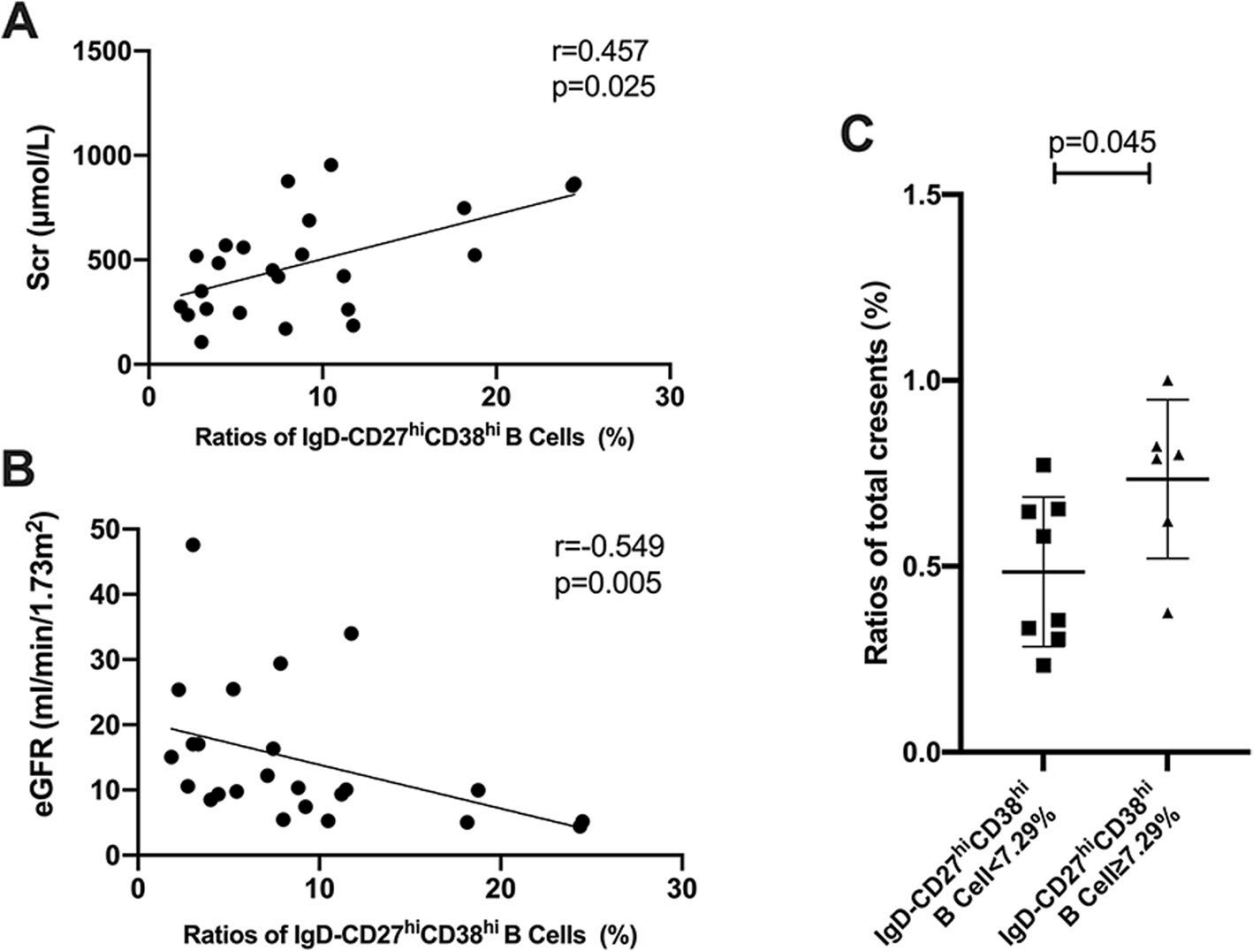
Association between frequency of IgD-CD27^hi^CD38^hi^ B cells and severity of renal involvement in patients with active AAV. **A** The frequency of IgD-CD27^hi^CD38^hi^ B cells correlated with Scr. **B** The frequency of IgD-CD27^hi^CD38^hi^ B cells correlated with eGFR. **C** The percentages of total crescents in renal biopsies were significantly higher in patients with ≥7.29% IgD-CD27^hi^CD38^hi^ B cells frequency than patients with <7.29% IgD-CD27^hi^CD38^hi^ B cells frequency

Among the 26 patients with AAV in active stage, associations between the frequency of IgD-CD27^hi^CD38^hi^ B cells and clinicopathological parameters were analyzed. Correlation analysis showed that the frequency of IgD-CD27^hi^CD38^hi^ B cells correlated with serum creatinine (Scr) (*r* = 0.457, *p* = 0.025, Fig. 3A) and estimated glomerular filtration rate (eGFR) (*r* = − 0.549, *p* = 0.005, Fig. 3B). No significant association between the frequency of IgD-CD27^hi^CD38^hi^ B cells and other clinical parameters was found, including ANCA titers, C-reactive protein (CRP), erythrocyte sedimentation rate (ESR), and BVAS.

We then divided the patients with active AAV into two groups based on the median frequency of IgD-CD27^hi^CD38^hi^ B cells, i.e., < 7.29% and ≥ 7.29%. The percentages of total crescents in renal biopsies were significantly higher in patients with a high IgD-CD27^hi^CD38^hi^ B cells frequency than in patients with a low IgD-CD27^hi^CD38^hi^ B cells frequency (73.5% ± 21.3% vs. 48.5% ± 20.1%, *p* = 0.045, Fig. 3C).

## Discussion

In the current study, we demonstrated an increase of circulating IgD-CD27^hi^CD38^hi^ B cells in AAV patients in active stage compared with patients in remission and healthy donors, and the frequency of IgD-CD27^hi^CD38^hi^ B cells correlated with Scr, eGFR, and percentages of total crescents in renal biopsies. This is the first study to describe the frequency of IgD-CD27^hi^CD38^hi^ B cells and its association with the severity of renal involvement in AAV patients, indicating a role of IgD-CD27^hi^CD38^hi^ B cell subset in the development of AAV, in particular, in renal involvement of AAV.

The most interesting finding in the current study is the association between IgD-CD27^hi^CD38^hi^ B cells and renal involvement of AAV patients, which might be consistent with some previous studies. Brix et al described B cell infiltrates in kidney biopsies from active ANCA-associated glomerulonephritis patients and found an association of organized lymphocytic infiltrates in their biopsy with renal failure, but not with tubular atrophy and interstitial fibrosis [27]. In the abovementioned study by von Borstel et al, the researchers demonstrated an increased frequency of CD27 + CD38^hi^ B cells in the kidney and urine, but not in the circulation, of GPA patients with active renal involvement, while patients without active renal involvement did not present with B cells in the urine, indicating that CD27 + CD38^hi^ B cells migrated from the circulation to the inflamed kidney. By further comparing the B cell subset distribution in blood and urine samples, they assumed that the migration of CD27 + CD38^hi^ B cells to the kidney depended on an active way rather than leakage into the urine [19]. Considering that IgD-CD27^hi^CD38^hi^ B cells are likely the direct precursors of autoantibody-producing plasma cells [17], the locally enriched specific active B cells might contribute to tissue destruction and organ dysfunction in inflamed kidney. Therefore, the renal B cell niche needs to be further studied in AAV.

In the current study, we did not find a significant correlation between the frequency of IgD-CD27^hi^CD38^hi^ B cells and ANCA titers in AAV patients, mainly MPO-ANCA, for there is a striking preponderance of MPO-ANCA rather than PR3-ANCA in Chinese patients with AAV [28]. One likely explanation might be that, besides antibody production by terminally differentiated B cells (plasma cells), B cells also contribute to disease development and progression by antibody-independent mechanisms, via serving as antigen-presenting cells which enhance T lymphocyte-mediated immunity or producing inflammatory cytokines such as interleukin-6 and tumor necrosis factor to reduce the anti-inflammatory activity of regulatory T cells and increase the differentiation of effector T cells [29]. After all, there is a disadvantage of ANCA level as a biomarker is that it is not fully reliable in all patients [30, 31], which may be attributed to the paratope, glycosylation, or other features of ANCA. That highlights the need for better indicators of disease activity, such as a specific subset of B cells which can be easily detected by flow cytometry.

Last but not the least, in order to better understand and assess the autoreactive B cells in autoimmune disease by newer approaches, such as functional autoreactive, flow cytometric, and single-cell cloning assays [32], it is crucial to identify and sort out the autoreactive B cells. Even though researchers developed a flow cytometry-based method to identify circulating PR3-specific B cells based on the specificity of their B cell receptor (BCR) targeted with tagged recombinant PR3 in patients with PR3-AAV, they also stated that the study of PR3-specific B cells might be easier than other autoantigen-specific B cells because PR3 is a very well characterized protein of moderate size (29 kDa), allowing the use of the whole protein as antigen source [33], which was in line with our pilot study aiming at identifying MPO-specific B cells using intact recombinant MPO (unpublished data). However, as abovementioned, MPO-ANCA is much more common than PR3-ANCA in Chinese patients with AAV [28], so it is still of great importance to identify the autoreactive B cells in MPO-AAV, while the IgD-CD27^hi^CD38^hi^ B cell subset could be considered as a candidate which needs further investigation.

## Conclusions

In conclusion, our study reports an increase of circulating IgD-CD27^hi^CD38^hi^ B cells and its association with the severity of renal involvement in patients with active AAV, as it is possible that IgD-CD27^hi^CD38^hi^ B cells are involved in the pathogenesis of AAV, especially the renal involvement of AAV. Our data might provide a new access to disease monitoring and pathogenesis investigation of AAV.

## Supplementary Information

**Additional file 1: Supplementary Table**. Interventions and outcomes of 8 patients with sequential blood samples of both active stage and remission.

## Data Availability

Data collection has been conducted in accordance to local regulation. The authors declare their availability in providing data if requested by the referees or the editorial team of the journal.

## Abbreviations

ANCA: Antineutrophil cytoplasmic antibody
AAV: ANCA-associated vasculitis
GPA: Granulomatosis with polyangiitis
MPA: Microscopic polyangiitis
EGPA: Eosinophilic granulomatosis with polyangiitis
Ig: Immunoglobulin
MPO: Myeloperoxidase
PR3: Proteinase 3
SLE: Systemic lupus erythematosus
CHCC: Chapel Hill Consensus Conference
CTX: Cyclophosphamide
BVASBVAS: Birmingham Vasculitis Activity Score
ELISA: Enzyme-linked immunosorbent assay
Scr: Serum creatinine
eGFR: Estimated glomerular filtration rate
BCR: B cell receptor
SD: Standard deviation
