# Supplementary material for: Increased frequency of IgD-CD27hiCD38hi B cells and its association with the renal involvement in ANCA-associated vasculitis
Source: Arthritis Res Ther. 2022 May 14;24:109. doi: 10.1186/s13075-022-02796-9 (PMC9107193; doi:10.1186/s13075-022-02796-9)
Supplement: Supplementary file 1 — Additional file 1: Supplementary Table. Interventions and outcomes of 8 patients with sequential blood samples of both active stage and remission. [file 13075_2022_2796_MOESM1_ESM.docx]

**Supplementary Table. Interventions and outcomes of** **8 patients with sequential blood samples of both active stage and remission**

| No. | Age/sex | Interventions | Outcomes |
| --- | --- | --- | --- |
| 1 | 56/Male | plasmapheresis, oral prednisolone and intravenous CTX | Partial remission |
| 2 | 66/Female | plasmapheresis, intravenous methylprednisolone pulse therapy, oral prednisolone and intravenous CTX | Partial remission |
| 3 | 54/Male | oral prednisolone and intravenous CTX | Complete remission |
| 4 | 58/Female | intravenous methylprednisolone pulse therapy, oral prednisolone and intravenous CTX | Complete remission |
| 5 | 85/Female | oral prednisolone | Partial remission |
| 6 | 77/Male | intravenous methylprednisolone pulse therapy and oral prednisolone | Treatment failure，ESRD |
| 7 | 63/Male | plasmapheresis, intravenous methylprednisolone pulse therapy, oral prednisolone and intravenous CTX | Partial remission |
| 8 | 63/Male | plasmapheresis, intravenous methylprednisolone pulse therapy, oral prednisolone and intravenous CTX | Complete remission |

[Abbreviations] CTX, cyclophosphamide
